# The complete mitochondrial genome of a leaf roller, *Eudemis lucina* (Lepidoptera: Tortricidae)

**DOI:** 10.1080/23802359.2019.1623729

**Published:** 2019-07-10

**Authors:** Jialiang Zhuang, Yongyan Li, Wenxu Yang, Weixing Feng, Mengfei Cao, Haili Yu

**Affiliations:** a Shaanxi Key Laboratory for Animal Conservation, Northwest University, Xi’an, P. R. China;; b College of Life Sciences, Shaanxi Normal University, Xi’an, P. R. China

**Keywords:** *Eudemis lucina*, Tortricidae, mitochondrial genome

## Abstract

The leaf roller, *Eudemis lucina*, is a potential pest of *Quercus* in East Asia. In this study, we described the complete mitochondrial genome of this species by high-throughput sequencing. The mitochondrial genome is found to be a circular molecule of 16,056 bp in length, which consisted of 13 protein-coding genes (PCGs), 22 tRNA genes, 2 rRNA genes, and a non-coding control region (A + T-rich region). The A + T content is 80.5% for the whole mitogenome. All PCGs are initiated by ATN codons, except for COI which is initiated by the CGA codon. Eight PCGs use a typical stop codon of TAA, whereas the remaining PCGs use incomplete stop codon of T–– or TA–. The non-coding control region is 1013 bp and located between s-rRNA and Met-tRNA.

*Eudemis lucina* belongs to the family Tortricidae (Lepidoptera) and is native to the East Asian continent (Byun et al. 1998; Liu and Li 2002; Oku 2005). This species is a potential pest of *Quercus*. The annual number of individuals caught by a light trap reached more than 1000 in successive years at Morioka in Japan (Oku 2005). In China, *E. lucina* is widely distributed (Li 2011), and we found it occurred in a great abundance in *Quercus* forests in Qinling Mountain. In this study, we assembled and characterized the mitochondrial genome of this species. The samples were collected from Qinling Mountain (33°24′N, 107°30′E), China, preserved in ethanol and stored in the insect specimen room of Northwest University.

Total genomic DNA was extracted from the muscle tissue using a DNA Extraction Kit (Tiangen Biotech, Beijing, China), and the complete mitochondrial genome was sequenced by high-throughput sequencing technology. The sequences of PCGs and rRNAs were identified using the NCBI BLAST function, then aligned with other lepidopteran sequences by applying MEGA 6.0 (Tamura et al. 2013). The tRNAs were confirmed by using tRNAscan-SE (Lowe and Eddy 1997). The map of the *E. lucina* mitochondrial genome was created by CG View – Circular Genome Viewer (Stothard and Wishart 2005). GC skew was measured using the following formula: GC skew = (G – C)/(G + C) (Perna and Kocher 1995). The circular mitogenomic sequence was deposited in GenBank under the Accession Number MK820027.

The complete mitochondrial genome of *E. lucina* is 16,056 bp in size, and contains 13 protein-coding genes (PCGs), 22 tRNA genes, 2 rRNA subunit genes (12s rRNA and 16s rRNA), and a control region (A + T-rich region). It has a base composition of A 40.6%, T 39.9%, C 11.6%, and G 7.8%. The AT content of the mitochondrial genome in *E. lucina* is approximately 80.5%. All PCGs are initiated by ATN codons (ATA ATT ATC ATG), while COI is initiated by the CGA codon. Eight PCGs use a typical stop codon of TAA, while the remaining PCGs use incomplete stop codon of T–– or TA–. Seventeen of tRNA can be found in tRNA scan-SE Search Server and the rest have no record. The length of 16s rRNA (l-rRNA) and 12s rRNA (s-RNA) are 1407 and 780 bp. The control region (A + T-rich region) between s-rRNA and Met-tRNA is 1013 bp. There are 18 intergenic spacer regions ranging from 1 to 46 bp in size, made up of a total length of 215 bp; the largest spacer (46 bp) resides between Gln-tRNA and ND2. There are also 4 intergenic overlapping regions ranging from 1 to 8 bp in size, made up of a total length of 18 bp. Phylogenetic analysis was performed using the neighbour-joining method based on the complete mitochondrial genome from *E. lucina* and other 17 leaf rollers. The result showed that *E. lucina* was clustered with eight Olethreutinae species (Figure 1), which was consistent with the conventional classification.

**Figure 1.**
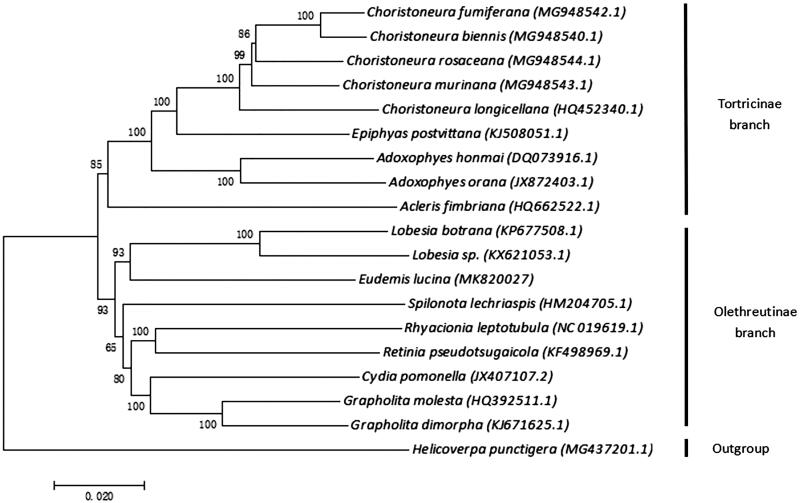
Phylogenetic tree showing the relationship between *Eudemis lucina* and 17 other leaf rollers based on the neighbour-joining method. *Helicoverpa punctigera* was used as an outgroup. GeneBank accession numbers of each species were listed in the tree.
